# Noninvasive Electromagnetic Wave Sensing of Glucose

**DOI:** 10.3390/s19051151

**Published:** 2019-03-07

**Authors:** Ruochong Zhang, Siyu Liu, Haoran Jin, Yunqi Luo, Zesheng Zheng, Fei Gao, Yuanjin Zheng

**Affiliations:** 1 School of Electrical and Electronic Engineering, Nanyang Technological University, Singapore 639798, Singapore; rzhang009@e.ntu.edu.sg (R.Z.); sliu023@e.ntu.edu.sg (S.L.); hrjin@ntu.edu.sg (H.J.); luoy0025@e.ntu.edu.sg (Y.L.); zesheng001@e.ntu.edu.sg (Z.Z.); 2 School of Information Science and Technology, ShanghaiTech University, Shanghai 201210, China

**Keywords:** noninvasive glucose measurement, IR spectroscopy, Raman spectroscopy, photoacoustic spectroscopy, THz spectroscopy, microwave sensing

## Abstract

Diabetic patients need long-term and frequent glucose monitoring to assist in insulin intake. The current finger-prick devices are painful and costly, which places noninvasive glucose sensors in high demand. In this review paper, we list several advanced electromagnetic (EM)-wave-based technologies for noninvasive glucose measurement, including infrared (IR) spectroscopy, photoacoustic (PA) spectroscopy, Raman spectroscopy, fluorescence, optical coherence tomography (OCT), Terahertz (THz) spectroscopy, and microwave sensing. The development of each method is discussed regarding the fundamental principle, system setup, and experimental results. Despite the promising achievements that have been previously reported, no established product has obtained FDA approval or survived a marketing test. The limitations of, and prospects for, these techniques are presented at the end of this review.

## 1. Introduction

**Figure 1:**
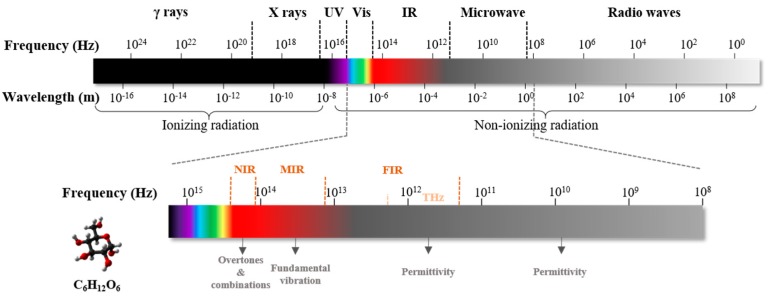
A diagram of the electromagnetic (EM) spectrum and its relevance to glucose. NIR, near-infrared; MIR, mid-infrared; FIR, far-infrared.

Blood glucose level is one of the most important physiological parameters that is associated with the metabolic and homeostatic mechanism in the human body. Diabetes mellitus is a prolonged metabolic disorder due to insufficient insulin production or an improper cell response to insulin [1]. The diabetic population was projected to be 552 million by 2030, as reported by David R. Whiting et al. [2], based on data sources for 80 countries. Diabetes imposes a heavy economic burden on patients and their families. According to the American Diabetes Association, the total cost associated with diabetes in the U.S. was $327 billion in 2017 [3]. Diabetes can be classified into three main categories: Type I, Type II, and gestational diabetes [4]. The first one is usually called “juvenile diabetes”, which is mainly diagnosed in children and results from a lack of insulin produced by beta-cells [5,6,7]. Only 5~10% of diabetic patients have this form. The second one is the most prevalent among diabetic patients, accounting for more than 90% [8], and is primarily caused by an unhealthy lifestyle and genes [9]. It is characterized by insulin resistance and sometimes combines with reduced insulin secretion [4,10,11,12]. The last category usually occurs among pregnant women and will either disappear or develop to Type II diabetes after delivery [4,13,14]. Long-term abnormal levels of glucose (hyperglycemia when the glucose level is >200 mg/dL [15] and hypoglycemia when the glucose level is <70 mg/dL [16]) often lead to complications, including accelerated atherosclerosis [17], stroke [18], neuropathy [19], nephropathy [20], and retinopathy [21]. In addition, it has been reported that diabetes also significantly increases the risk of cause-specific death [18,22,23]. Since there is no specific cure for diabetes [24], daily glycemic measures need to be carried out together with active treatments (insulin injection or bariatric surgery) to improve diabetic patients’ quality of life. Since 1962, when Clark and Lyons proposed the electrochemical method [25], glucose oxidase (GOx) has been widely applied for glucose determination. The well-established glucose meters are mainly based on electro-enzymatic reactions, which require a finger-prick device to obtain a drop of blood (~1 μL) and apply it onto a disposable testing strip [26,27]. Although the accuracy of this kind of invasive device has been proved and is accepted, physical pain and infection can be easily caused [28,29] with a discrete readout only. Moreover, the annual cost of testing strips is estimated to be $750 per patient [30]. Therefore, semi-invasive or minimally invasive devices have been developed with the aim of replacing those finger-prick devices and achieving continuous blood glucose monitoring (CBGM). They usually measure the glucose concentration in interstitial fluid (ISF) by implanting a tiny and relatively painless subcutaneous sensor. Nevertheless, the sensitivity gradually degrades as the protein builds up on the surface of the sensor, and hence frequent calibration is required [31,32]. Much effort has been devoted to the development of truly noninvasive glucose sensors that employ various emerging technologies. Among those methods, electromagnetic (EM) wave sensing has drawn much attention due to its rich interactions, including absorption, scattering, and transmission, with particular compounds inside the body. As shown in Figure 1, EM waves can be classified as radio waves, microwaves, terahertz (THz) waves, visible/infrared (IR) light, ultraviolet, X-rays, and gamma rays [33] based on different frequencies or wavelengths. EM waves with higher frequencies carry higher photon energy, which can damage tissues and organs by ionizing radiation. In contrast, non-ionizing radiation is relatively safe and suitable for noninvasive biomedical sensing and imaging. The boundary between ionizing and non-ionizing radiation falls in the UV region with a photon energy between 10 eV and 33 eV, which is not well-defined. Herein, we will mainly discuss the utilization of non-ionizing radiation, including vis/IR, T-ray, and microwaves, regarding the application of a noninvasive glucose measurement. A molecule’s characterization is realized by its specific vibrational frequencies. Fundamental vibrations can be excited by the mid-infrared (MIR) light, known as the “fingerprint region” of many molecules, including glucose. Overtones and combinational bands usually fall into the near-infrared (NIR) region, while T-waves and microwaves can be used to detect tissue permittivity variations caused by glucose fluctuations. Several review articles for minimally invasive/noninvasive glucose monitoring have been published previously. The authors in [32] focused more on noninvasive glucose monitoring devices as commercial products and briefly introduced those related techniques. The authors in [34] emphasized NIR spectroscopy with comprehensive descriptions, while the other techniques were just mentioned in brief. The authors in [35] and [36] presented recent developments in minimally invasive CBGM biosensors for body fluids testing. In this article, we will review the EM-based noninvasive glucose sensing techniques; more specifically, optical methods, photoacoustic spectroscopy, THz spectroscopy, and microwave sensing. We will focus more on the theoretical basis for these techniques, implementation details from scientific and engineering points of view, and updating the reader on recent achievements from different groups to tackle this challenging issue. Various EM-based technologies are listed and compared in Section 3. Section 4 contains a discussion on, and the outlook for, noninvasive glucose sensing.

## 2. Performance Evaluation

**Figure 2:**
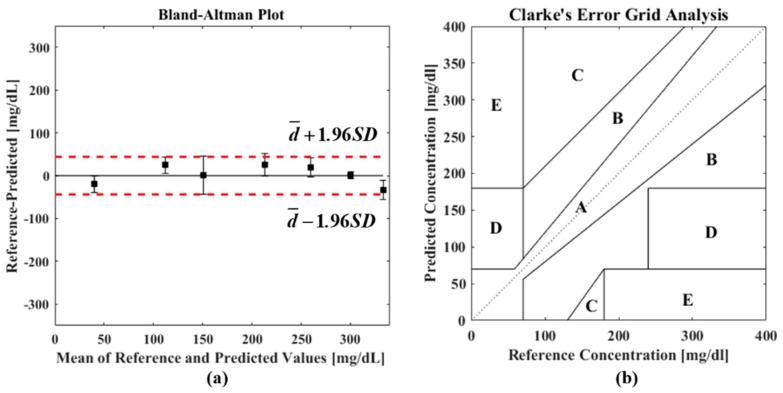
Examples of (**a**) a Bland–Altman plot and (**b**) a Clarke Error Grid (CEG) Analysis.

To evaluate the performance of noninvasive techniques and devices, the obtained data are usually calibrated and paired with references measured by invasive blood glucose meters at the same time point. Several indicators are often adopted to quantitatively assess the performance from a statistical and clinical point of view. Firstly, the coefficient of correlation *R* can be used to show the degree of correlation between two data sets. Its value always varies within ±1, where a positive value indicates the same variation trend while a negative one represents the opposite trend. Another indicator, the *R-squared* (*R*^2^) value, is known as the coefficient of determination, which measures the goodness of a linear regression. Besides these, the root-mean-square error (RMSE), the mean absolute error (MAE), and the mean absolute percentage error (MAPE) [37] are used to evaluate the deviation of predicted values compared to references. The limit of detection (LOD) defines the minimum amount of target sample that can be measured. It reflects the system sensitivity and noise performance. In 1986, J. M. Bland and D. G. Altman claimed that using a correlation is misleading and they suggested a new statistical approach to assess degree of agreement: the Bland–Altman plot [38,39], as shown in Figure 2a. It can be used to show the difference between measured values and references, where the solid black line represents their mean difference ($\bar{d}$) and the two dotted lines are “limits of agreement” whose values are calculated as $\bar{d}\pm 1.96SD$ (*SD* is the standard deviation of the differences). Apart from the abovementioned statistical accuracy evaluation methods, W. L. Clarke proposed the use of a scatterplot to describe the clinical accuracy of glucose meters, which has become the “gold standard” [40]. As shown in Figure 2b, the Clarke Error Grid (CEG) is divided into five regions, where A contains values within a ±20% deviation from the reference, and B contains predictions with >20% error but not leading to an inappropriate treatment. Data in these two regions are regarded as clinically acceptable. On the contrary, predictions falling into region C will lead to overcorrection of normal glucose levels and D represents a failure to detect abnormal glucose levels for prompt treatment. Data falling into region E will result in an erroneous and a dangerous treatment. Measurement results in these three regions (C, D, and E) are not beneficial in patients’ daily care.

## 3. Methodologies for Noninvasive Glucose Sensing Utilizing Electromagnetic Waves

In this section, various EM-wave-based noninvasive glucose monitoring techniques are reviewed in detail, including the basic theories, system instrumentation, and laboratory tests.

### 3.1. Infrared (IR) Spectroscopy

There are several kinds of interactions between light and biological tissues depending on the properties of target tissues and the characteristics of illuminating sources. The interactions can be mainly categorized into absorption, transmission, emission, reflection, and scattering. Among those, absorbance information is by far the most widely used. Molecules with vibrational and rotational motions tend to absorb light at matched frequencies or wavelengths due to resonance. More specifically, chemical bonds can move in the form of bending, symmetrical stretching, asymmetrical stretching, et al. [41]. The quantum vibrational energy bands usually fall into the infrared (IR) region [42]. Thus, IR spectroscopy has been widely applied in analytical chemistry for characterizing samples in various states such as gases, liquids and solids. Based on different excitation sources, it can be classified into near-infrared (NIR) spectroscopy [43], mid-infrared (MIR) spectroscopy [44], and far-infrared (FIR) spectroscopy [45]. In Section 3.1, we will discuss NIR and MIR, and separately discuss THz in Section 3.6, which overlaps with the FIR region.

#### 3.1.1. Near-Infrared (NIR) Spectroscopy

NIR radiation was discovered by Sir William Herschel in 1800 [46], and the first NIR spectrum was obtained in 1881 by Abney and Festing in the range of 1000–1200 nm [47]. Nowadays, NIR spectroscopy utilizes an EM wave in the range of 700–2500 nm [34,43,48,49], which covers several optical windows where photons have less interactions with interfering tissue compounds, such as water, hemoglobin, and lipids, so that the penetration depth can achieve several millimeters [32,50], where capillary beds locate [51,52,53]. The absorption in this wavelength range corresponds to a combination, the first overtone, the second overtone, or a higher-order overtone of a fundamental molecule’s stretching and bending [43,54,55]. Glucose is a kind of monosaccharide with the molecular formula C_6_H_12_O_6_ in the form of pyranose. It has several absorption peaks in the NIR region, which are listed in Table 1.

The attenuated light simply due to the absorption of analytes after they pass through tissue is governed by the Beer–Lambert Law, which is expressed as

(1) $$I=I_{0}\exp(-\mu_{a}l)$$

where *μ_a_* is the absorption coefficient and *l* is the effective optical path length. *μ_a_* is proportional to *εC* cm^−1^, where *ε* represents the molar extinction coefficient and *C* is the molar concentration of the analyte [56,61,62]. *μ_a_* may increase with an elevated glucose level due to its intrinsic absorption or decrease due to a water displacement effect. The latter is less specific as changes of other components can also result in the same effect [56].

The basic instrumentation of NIR spectroscopy consists of a light source, such as a tungsten halogen lamp, and an IR detector. Then, the analog signal is filtered and amplified before being digitized by an analog-to-digital converter (ADC). Given the complexity of interfering component matrices, various signal-processing techniques are adopted to extract glucose-related information, including principle component regression (PCR)-, partial least-square regression (PLSR)-, and artificial neural network (ANN)-based analysis [63,64,65,66,67]. 

**Figure 3:**
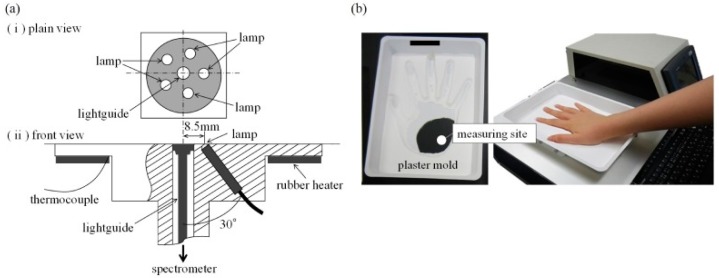
(**a**) Setup details. (**b**) A plaster mold to restrain a patient’s hand. Reprinted with permission from [68].

Uwadaira et al. identified the informative bands in the NIR region by 391 data sets from 34 participants’ 2-h carbohydrate tolerance tests [68]. Five tungsten halogen lamps at 1 W placed in a circle were used as an illuminating source and the diffusely reflected light was collected by the guide of a spectrometer. Patients’ hands were fixed by plaster molds. The detailed setup is shown in Figure 3. Wavelengths were scanned from 700 to 1050 nm at a step size of 1 nm. They suggested some characteristic bands (1018 nm, 1030 nm, and 1042 nm) for noninvasive glucose measurement, although the correct assignment and relation to glucose are hard to explain. Xue et al. compared linear and nonlinear regression methods by using PLS and ANN on living rats [69]. Besides this, different combinations of pretreatment methods, including first derivative, second derivative, and vector normalization, were also investigated. In their study, PLS achieved a better performance than ANN did with a lower RMSE and a higher R. Yang and co-workers investigated the informative bands in shortwave and first overtone regions [57] by a homemade NIR Fourier transform spectrometer. All-reflective optics with an Offner relay lens were employed to reduce axial chromatic and magnification chromatic aberrations. They reported that the characteristic peaks of glucose solution are 938 nm, 1040 nm, and 1295 nm caused by glucose variations in the short waveband. They also found a prominent absorption peak at 1639 nm in the first overtone band. Transmission spectra from the middle fingers of seven volunteers were obtained by performing an oral glucose tolerance test (OGTT), and characteristic peaks were only observed in the shortwave band due to the low signal-to-noise ratio (SNR) in the first overtone band.

NIR spectroscopy allows for deep tissue (>1 mm) measurement with high sensitivity. The instrumentation is simple and relatively low-cost. However, glucose absorption peaks are broad in this region and may overlap with other interferences, such as water and lipids. Besides this, temperature, humidity, and other environmental factors may also affect the measurement results.

#### 3.1.2. Mid-Infrared (MIR) Spectroscopy

Unlike NIR spectroscopy, MIR spectroscopy employs a longer wavelength that ranges from 2500 to 10,000 nm [70] where the well-known “fingerprint region” of glucose locates. The featured absorption peaks in this region are sharper and provide better specificity than NIR spectroscopy does. The maxima of glucose absorption in the MIR region are listed in Table 2.

Despite the relatively good specificity or selectivity, penetration depth is severely limited due to the strong absorption of water and lipids [79]. Thus, MIR light can only pass through the first layer of skin—stratum corneum—with a thickness of 10–20 μm [80] and detect glucose of ISF found in the stratum spinosum layer [74,81]. It was proved that ISF in epidermis has a strong correlation with blood glucose in spite of several minutes’ delay [82,83,84,85]. Thanks to the recent development of Quantum Cascade lasers (QCLs) with high power, several groups have reported in vivo applications of MIR spectroscopy [76,86,87,88,89,90]. Liakat et al. proposed a system with an external cavity QCL (EC-QCL) and a hollow core fiber to deliver light to the human palm. Then, backscattered light was collected by a fiber bundle and a mercury cadmium telluride (MCT) detector. PLSR and second derivative spectroscopy were applied for prediction in three human subjects. All data points fell in Region A and B of the CEG, which shows that the maximum error in the prediction results was within 20%. One-hour continuous measurement was also conducted, which showed the general trend of glucose variation successfully. Their group recently improved the system by adding an integrating sphere to enhance the collection efficiency of backscattered light [90]. Kino et al. adopted attenuated total reflection (ATR) spectroscopy and utilized the evanescent wave generated when total internal reflection occurs to penetrate into the sample. The absorption of the evanescent wave by the sample can be measured by an MCT detector to infer the glucose concentration. Their hollow optical-fiber-based spectroscopy system is equipped with a trapezoidal ATR prism that allows for multiple reflections to enhance the sensitivity [76]. Inner lip mucosa were selected as the measurement site owing to the relatively thin stratum corneum and the lack of a keratinized layer, which makes the ISF accessible to the evanescent wave. They found that an absorption peak at 1155 cm^−1^ was most relevant to glucose, and originated from its pyranose ring structure. An in-vivo experiment was conducted. The *R*^2^ value of 0.75 was achieved, and the all of the data points were in Region A of the CEG.

The selectivity of MIR spectroscopy is better than that of NIR spectroscopy as it covers glucose’s “fingerprint region”. The major drawback of this method is that the penetration depth is limited to several microns and only ISF can be reached. Besides this, the illuminating source, such as a QCL, is bulky and expensive.

### 3.2. Photoacoustic Spectroscopy

The photoacoustic (PA) effect refers to a phenomenon where an object absorbs heat from light and undergoes thermal expansion followed by the generation of an acoustic wave. Combining the high contrast of an EM wave with the deep penetration of an acoustic wave in biological tissue, the PA technique is able to achieve prominent performance in bio-sensing and bio-imaging applications. Although, theoretically, any kind of EM wave can generate a PA signal, vis/IR lasers are the most frequently reported due to the wide availability of sources, the convenience of manipulation, and the rich functionality. Compared to IR spectroscopic methods for glucose detection, PA spectroscopy collects acoustic waves, which are more immune to tissue scattering and directly related to the laser energy deposited in skin, yielding deeper penetration and better sensitivity. Besides pure optical properties, such as absorption, a PA signal also contains information about the mechanical or acoustic properties of the tissue [91,92], which could be related to glucose concentration. The received PA signal by an ultrasound transducer at position z can be expressed by a one-dimensional wave equation along the z-direction [93] and solved by Green’s function [94] as follows:

(2) $$p(z,t)=\frac{\beta v^{2}}{2C_{p}}\eta F\mu_{a}\delta (t-\frac{z}{v})$$

where *β* is the thermal expansion coefficient, *v* is the sound velocity, *C_p_* is the heat capacity at constant pressure, *η* represents the optic-heat conversion efficiency, and *F* represents the laser fluence. *μ_a_* and *v* vary with glucose concentration, which can be utilized in prediction [95,96,97]. Similar to optical spectroscopy, laser wavelengths ranging from NIR to MIR have been adopted in various studies [74,98,99,100,101].

**Figure 4:**
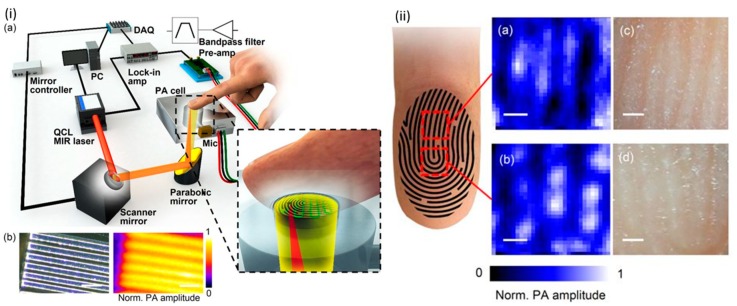
(**i**) (a) A schematic of the position scanning photoacoustic (PA) microscopy system and (b) system resolution evaluation by SU-8 (a kind of photoresist structure). (**ii**) PA images (a,b) and corresponding micrographs (c,d) of two fingertip regions. Reprinted with permission from [100]. PC, personal computer; QCL, Quantum Cascade Laser; DAQ, data acquisition.

Sim et al. combined an MIR PA sensor with a raster scan to investigate the microscopic structure of skin and reduce skin condition variation during measurement [100]. The system consists of an external cavity QCL (950–1240 cm^−1^) as the illuminating source and a photoacoustic cell, whose resonance frequency was designed to match the laser repetition rate. The PA signal was collected by a microphone, amplified by a pre-amplifier, and then sent to a lock-in amplifier. The index finger was in direct contact with the PA cell, and the resolution achieved was 90 μm. They suggested that the dark area between the friction ridges of a finger was non-secreting and immune to sebum and sweat, so better prediction results could be achieved there. The experimental setup and acquired images are shown in Figure 4.

**Figure 5:**
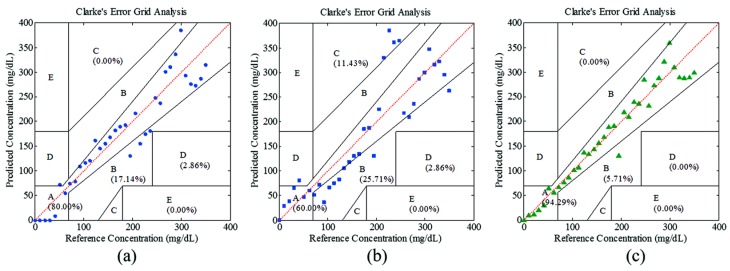
Correlations between references and predicted glucose concentrations shown in CEGs by using (**a**) the PA amplitude, (**b**) the time delay, and (**c**) a data fusion. Reprinted with permission from [101].

Zhang and co-workers proposed to utilize both PA signal amplitude and time information to enhance prediction accuracy by data fusion, without increasing apparatus and system complexity [101]. They employed an NIR laser at ~1600 nm, which is one of the broad glucose absorption peaks. Glucose solutions at both high and low concentrations were tested, and the prediction accuracy was significantly enhanced by data fusion compared to single-parameter-based prediction. The prediction results on a glucose solution with a concentration in the physiological range (0–400 mg/dL) were evaluated by a CEG and are shown in Figure 5. They also proposed a “guide-star”-assisted indirect method to enhance the sensitivity by combining [102] the fundamental principle of transmission NIR spectroscopy with that of PA spectroscopy. The modified expression of PA pressure generated by the “guide star” is

(3) $$p(z,t)=\frac{\beta v^{2}}{2C_{p}}\eta \mu F_{0}exp[-\varepsilon (\lambda )Cl]\delta (t-\frac{z}{v})$$

where *l* is the adjustable optical path length used to amplify the signal difference caused by glucose variation. To verify the method, 1-mm- and 2-mm-thick quartz cuvettes were used. The latter showed better sensitivity and accuracy as expected. This method provides an alternative solution to the noninvasive PA measurement of glucose by detecting a transmitted signal passing through the target tissue with a certain thickness (the optical path length).

Photoacoustic technology detects a laser-induced pressure change by an acoustic transducer with good sensitivity. The acoustic wave is less-scattered in tissue, resulting in a better penetration depth. Nevertheless, the measurement is sensitive to ambient pressure, temperature, and humidity.

### 3.3. Fluorescence Spectroscopy

Fluorescence refers to a phenomenon in which substances emit light (usually at a longer wavelength) when they absorb EM radiation. Fluorescence spectroscopy is useful in medical and biochemical analysis of chemical groups. The excitation source usually falls in the ultraviolet (UV) region with high photon energy. Fluorescence has the advantage of high sensitivity, which even allows for single molecule detection [103]. In addition, the characteristic emission of a certain fluorophore also guarantees high specificity. Fluorescent methods for glucose measurement can be based on intrinsic skin fluorescence spectroscopy (SFS) or specially designed molecule reporters. For the design of extrinsic fluorophores, several factors, including quantum yield, photostability, absorption, and wavelength, have to be considered [104]. Both fluorescence intensity and lifetime can be evaluated to provide sufficient information.

**Figure 6:**
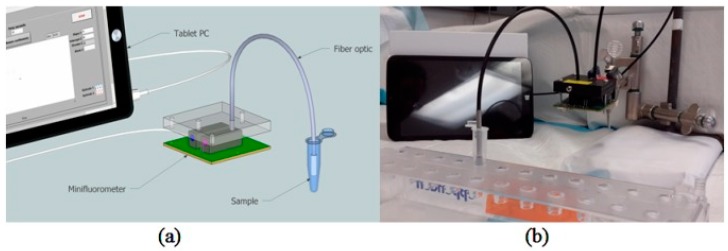
(**a**) A schematic of the fiber optic sensor system. (**b**) In vitro experimental setup. Reprinted with permission from [105].

VeraLight, Inc. announced an SFS-based product, SCOUT DS, which has obtained market approval in several countries. It aims to alert adults who are at risk of diabetes by identifying advanced glycosylation end products (AGEs). Glucosense, developed by Prof. Jose’s group from the University of Leeds, utilizes ion-doped silica glass, whose fluorescent lifetime depends on the glucose concentration. They claimed that blood glucose can be measured by simply placing a finger on the glass. Tiangco and co-workers fabricated a non-enzymatic fiber optic sensor based on a glucose binding protein (GBP), aiming to detect a tiny amount of passively diffused glucose though the skin [105]. The system consists of a fiber with a GBP at the tip, a mini-fluorimeter, and other electronics, including a driver, lock-in photodetectors, and a controller as shown in Figure 6. The target was excited at 405 nm and fluorescence at 540 nm was collected. An in-vitro experiment verified the linearity of the sensor from 4 to 20 μM with a sensitivity of 0.1296 μM^−1^. A phantom experiment was conducted with porcine skin and a static Franz cell to demonstrate passive diffusion. Because of the thickness of 700 μm, the measurement could only be realized when the glucose in the bottom reservoir is >8 mM.

Su et al. designed a sensor based on glucose oxidation by indirectly sensing oxygen consumption [106]:

$$Glu\cos e+O_{2}+H_{2}O\overset{GO_{X}}{\to }Gluconic\;Acid+H_{2}O_{2}.$$

Glucose oxidase was attached on an oxygen-sensing film, and the fluorescence intensity was correlated with the glucose concentration. The used excitation wavelength was 380 nm, and a 650-nm emission was received. The sensing specificity and reversibility were well-proved by a seven-day in-vitro test, suggesting its potential to be used as a tear glucose sensor.

Fluorescence-based methods have excellent sensitivity. However, the excitation source, which falls in the UV region, suffers from strong scattering and it usually measures body fluids, such as tears and sweat, which have relatively poor correlation with blood glucose.

### 3.4. Raman Spectroscopy

**Figure 7:**
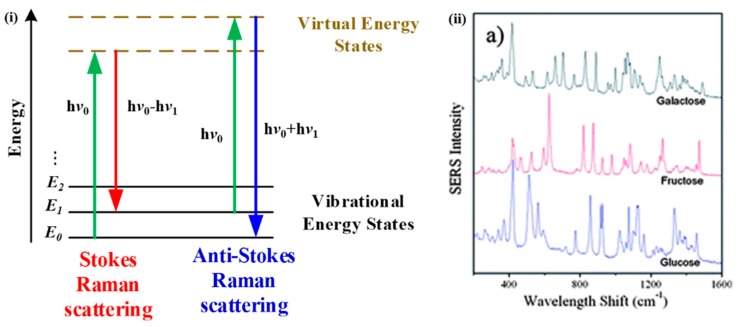
(**i**) An energy diagram of Raman scatterings. (**ii**) Examples of Raman spectra of different sugars. Reprinted with permission from [108]. SERS, surface-enhanced Raman spectroscopy.

Raman spectroscopy relies on the inelastic scattering of photons, named after C.V. Raman [107], to identify different molecules. There are two types of Raman scattering: stokes scattering, in which incident photons transfer energy to molecules, resulting in scattered photons with lower energy; and anti-Stokes scattering, which leads to increased photon energy when molecules transfer energy to incident photons, as illustrated in Figure 7i. A Raman spectroscopy system consists of a coherent and monochromatic light source, a grating to disperse the light, filters, and a photodetector to obtain Raman spectra. Several advanced Raman techniques have been developed, such as surface-enhanced Raman spectroscopy (SERS), resonance Raman spectroscopy (RRS), tip-enhanced Raman spectroscopy (TERS), and their combinations. Glucose has characteristic scattering features in the range of 400–1500 cm^−1^ as shown in Figure 7ii [108].

Enejder and co-workers successfully demonstrated the first application of Raman spectroscopy for noninvasive glucose monitoring [109] on 17 volunteers. Four hundred and sixty-one spectra were collected and compared with a reference glucose level. A good correlation (*R*^2^ = 0.87) was obtained, and the average prediction error was 7.7%. Zheng et al. developed a miniaturized wearable Raman spectroscopy system [110]. The main body consists of a miniature Raman spectrometer, a 785-nm laser diode as an illuminating source, lenses, a Czerny–Turner optical system, and a linear array charge coupled device (CCD). The sensor probe was made of a wearable fiber with a thallium-doped grin lens for interaction and collection. The signal peak area was evaluated with a nonlinearized PLS model to predict the glucose level. Raman spectra were examined from 300 to 3000 cm^−1^. Ten volunteers were tested, and the mean *R*^2^ value was found to be 0.844, suggesting the feasibility of this system for clinical application.

Owing to the sharp and distinct peaks, Raman spectroscopy has excellent selectivity. However, the long acquisition time makes it sensitive to laser intensity fluctuation, and the signal is weak.

### 3.5. Optical Coherence Tomography (OCT)

OCT is able to provide depth-resolved information on skin layers by detecting coherently backscattered photons. It was developed by Huang et al. in 1991 [111]. The system usually consists of a two-beam interferometer and a photodiode. An envelope of an interferometric signal can be used to reconstruct a cross-sectional, two-dimensional (2D) image and the light attenuation information can be calculated thereafter. Noninvasive glucose measurement by OCT is based on the fact that glucose variation in extracellular fluid (ECF) will induce a refractive index mismatch change between ECF and cellular components. Thus, the scattering coefficient will change, which is reflected by the backscattered signal strength. The effect of the refractive index mismatch induced by glucose is higher than that of other body osmolytes [112].

Pretto and co-workers presented a method based on both spatial information on light’s total attenuation coefficient and a temporal analysis of the speckle decorrelation time to predict glucose level [113]. The former is due to a refractive index mismatch and the latter is because of a viscosity change caused by glucose fluctuation. A mouse blood sample with a glucose concentration from 160 to 310 mg/dL at a step size of 50 mg/dL was tested. An increase in signal attenuation was shown with increasing glucose concentration, which resulted from an osmotic shock of red blood cells (RBCs) that only occurred in vitro. The data were well-fitted by a linear model with an *R*^2^ of 0.98. Besides this, they also suggested that the decorrelation time of the Brownian motion is proportional to viscosity. An autocorrelation analysis was performed, and the apparent linear trend between glucose concentration and average decorrelation time was shown. Lan et al. applied OCT to diabetic patients and showed that the monitoring results were better than those of healthy subjects [114] based on *R* values (0.91 for diabetic patients and 0.78 for healthy volunteers).

OCT provides good resolution; however, it is similar to the scattering method for noninvasive glucose sensing. The obtained value can be affected by the presence of other interfering components, which results in poor selectivity.

### 3.6. Terahertz Spectroscopy

EM waves with frequencies ranging from 0.3 to 3 THz are named terahertz waves, which fall between far-infrared and microwaves. They are sensitive to not only the molecule itself but also intra- and inter-molecular rotational and vibrational transitions [115]. The excitation sources include, but are not limited, to QCLs, Gunn and tunnel transit-time (TUNNETT) diodes, solid-state electronic devices, such as uni-travelling-carrier photodiodes (UTC-PDs), and optical rectification. Photoconductive antenna, Schottky barrier diodes (SBDs), and bolometers have been widely used as terahertz detectors [116]. Moreover, electro-optic (EO) techniques can be also employed with nonlinear crystal [117]. Terahertz time-domain spectroscopy (THz-TDs) was developed for the characterization of chemical compounds and medical imaging by non-ionizing radiation. Both amplitude and phase information can be obtained in the time domain. Although many nonmetallic or nonpolar materials are transparent to T-rays, the penetration depth is limited to hundreds of micron in biological tissues, due to the strong water absorption [118,119]. Hence, transmission spectroscopy can be only applied to ex vivo sample testing [120] and is not suitable for in-vivo applications. Similar to MIR spectroscopy, reflectance spectroscopy is usually considered for ISF testing.

Cherkasova et al. adopted an ATR optical scheme with a silicon prism to obtain THz spectra of human skin [121]. The T-wave was emitted by a femtosecond laser and a low temperature grown gallium arsenide (LT-GaAs) surface, and the reflected wave was detected by a 1-mm-thick EO crystal. A 0.1–2.5 THz frequency range was used. Six volunteers were measured for 90 min with OGTT. They explained the dielectric permittivity change in the solution by a Debye model, which involves the dielectric constant and the relaxation time. When water molecules are bound to glucose molecules, the relaxation time of the bound water will change and affect the spectra shape. However, only qualitative trends were observed and explained in the article. Chen and co-workers quantified blood glucose level through ex-vivo experiments using THz-TDs [120]. A 0.2–0.9 THz wave was generated by an indium arsenide (InAs) wafer and an 800-nm femtosecond laser. The system was arranged in transmission mode. Twenty fresh blood samples from diabetic patients were measured and compared with a glucose meter. A linear correlation was obtained and the relative error was less than 15%, which verified the capability of THz-TDs to quantify glucose level.

THz is sensitive to molecules and intra/inter-molecule transitions. Nevertheless, it requires a dedicated excitation source and suffers from poor penetration depth, which means that a T-wave can only reach ISF with a reflection setup.

### 3.7. Microwave Sensing

A microwave is an EM wave that ranges from 1 mm to 1 m, which corresponds to frequencies between 300 GHz and 300 MHz. Microwaves can easily penetrate homogeneous tissue with a thickness of millimeters, especially in a low-frequency range [122], which remains a challenge for most of the optical-based methods. Besides this, the cost of microwave sensors is usually low and fabrication is relatively easy. The reflection, transmission, and absorption of a millimeter wave are closely related to the dielectric property or relative permittivity of skin [123,124], which varies with glucose fluctuations [125,126,127]. The complex permittivity varies with frequency, and can be expressed by the Cole–Cole equation [128]:

(4) $$\varepsilon^{*}(\omega )=\varepsilon_{\infty }+\frac{\varepsilon_{s}-\varepsilon_{\infty }}{1+{\left(j\omega \tau \right)}^{1-\alpha }}$$

where $\varepsilon_{\infty }$ and $\varepsilon_{s}$ are dielectric constants at infinite and static frequencies, respectively, and τ is a relaxation time constant. These three parameters are related to glucose concentration [129]. α is a value between 0 and 1. Scattering or S parameters describing the two-port networks are usually investigated to infer glucose change. In-vitro (with a tiny sensing volume (~nL)) as well as in-vivo applications utilizing a microwave for glucose measurement have been studied recently [130,131,132,133,134,135,136,137,138,139], and we will mainly focus on noninvasive detection here.

Xiao and Li proposed an ultra-wide band (UWB) microwave-based method using a pair of planar antennas applied to an earlobe [133]. A tissue-mimicking phantom with fat, blood, and skin layers was made to model the earlobe. A short-time Fourier Transform (STFT) was applied for the time-frequency analysis. A glucose concentration from 0 to 400 mg/dL with a step size of 50 mg/dL was tested. The regularity of forward gain S_21_ verified the sensor function at 6.5 GHz. Similarly, Saha and co-workers presented microstrip antennas operating at 60 GHz and measured S_21_ to predict glucose levels [137]. They demonstrated the sensor’s performance by in vitro and in vivo OGTT. The detection limit for aqueous glucose solution was found to be 1.33 mmol/L (1 mmol/L = 18 mg/dL), which is far below the physiological range. Apart from the amplitude of S-parameters, other characteristics of an equivalent radio frequency (RF) circuit can also be utilized to reflect permittivity change due to glucose fluctuation. Choi et al. designed a split-ring resonator that aimed to eliminate the temperature effect for noninvasive and continuous glucose monitoring [137,139]. The ring nearest to the measurement site is responsible for interacting with tissue, whereas the ring that is furthest away acts as a reference resonator. The two rings are made of silver-coated copper wire and exhibit similar temperatures. OGTT was carried out, and the 3-dB bandwidth changes in the resonance peaks were measured and correlated with a reference glucose concentration. One hundred percent of data points fell within Zone A and B of the CEG.

Microwave-based glucose sensors are low-cost and can be easily miniaturized. The penetration depth is also appreciated, especially at a low-frequency range. However, it lacks selectivity as the dielectric constant is strongly affected by other blood components as well.

## 4. Discussion and Conclusions

Researchers have not stopped pursuing the ultimate solution for noninvasive blood or ISF glucose monitoring, driven by the tremendous academic and market values. EM-wave-based methods are the most attractive ones owing to their wide spectral region and being information-rich. We listed several representative techniques herein and the corresponding achievements to date. Although a number of groups have demonstrated in-vitro and in-vivo applications, there is no well-recognized method that has conquered the great difficulty so far. Global challenges include sensitivity, specificity, system stability, and calibration. For example, IR and PA spectroscopy often rely on a powerful light source with a wide wavelength range as well as advanced calibration methods, such as PCR and ANN, to achieve specificity. Moreover, the penetration depth is also limited due to the strong tissue absorption in this region. Microwaves can reach deeper tissue, but there is no specific absorption for glucose. In other words, they lack specificity. Raman spectroscopy possesses favorable specificity. Nevertheless, its sensitivity is poor and it is sensitive to laser stability. Fluorescence-based methods have prominent sensitivity and specificity; however, most of them require exogenous markers and are not truly noninvasive or measure such body fluids as tears. To summarize, Table 3 that briefly compares different EM-wave-based methods is presented below.

A quantitative performance comparison is listed in Table 4, where the parameters representing each technology were extracted from the literature discussed in Section 3.

For clinical application and device development, environmental factors, such as temperature, humidity, pressure, and movement, have to be taken into account during measurement. Besides this, effort should also be devoted to miniaturization and cost reduction of the instrumentation to convert these technologies into commercial products, since portability and price are the major concerns of end-users, which determine the potential market shares. Based on the above considerations, NIR spectroscopy, PA spectroscopy, fluorescence, Raman spectroscopy, and microwave sensing have great potential to be turned into affordable products and used in daily healthcare. There are several tentative products employing the abovementioned technologies, such as Glucotrack, Glucosense, Aprise, and SugarTrac. Despite that, researchers are continuing to explore unknown areas and promising results have been reported. The requirements have yet to be met, and there is still a long way to go for these novel approaches to replace the current finger-prick glucose meters.

## Figures and Tables

**Table 1:** Absorption peaks of glucose in the NIR region and the corresponding functional groups.

No.	Wavelength (nm)	Functional Group
1	2273	Combination of O-H/C-O stretching [55]
2	2261	νCH + νCCH [56]
3	1688	2νCH [56]
4	1638	First overtone [57]
5	1536	νOH + νCH [56]
6	1408	2νOH [56]
7	1126	3νCH [56]
8	1042	Combination of νCH [58,59]
9	1018	Combination of νCH [60]
10	939	3νOH [56]
11	930	3νCH_2_ [58]
12	910	4νCH [58,59]

**Table 2:** Absorption peaks of glucose in the MIR region and the corresponding functional groups.

No.	Wavelength (nm)	Functional Group
1	8000	C-H bending vibrations [70,71,72,73]
2	8244	[74]
3	8658	Pyranose ring [75,76]
4	8680	[77]
5	9290	C-H bending vibrations [70,71,72,73,74,77]
6	9551	C-H bending vibrations [70,71,72,73]
7	9680	ν(C–O–H) or ν(C–O–C) vibration [72,73,74,77,78]
8	9746	C–O–H bending vibration [70,72,73]

**Table 3:** Comparison of different EM-wave-based methods.

Technology	Penetration	Target	Sensitivity	Selectivity	System Size	Cost
**NIR**	>1 mm	ISF, blood	High	Good	Portable	Low
**MIR**	Several μm	ISF	High	Good, better than NIR spectroscopy	Large	High
**PA**	Better than IR spectroscopy	ISF, blood	High	Good	Portable	Low
**Fluorescence**	<1 mm	Tear, ISF, blood	High	Excellent	Portable	Low
**Raman**	<1 mm	ISF, tears	Low	Excellent	Portable	Medium
**OCT**	<1 mm	ISF	High	Poor	Portable	Medium
**THz**	~100 μm	ISF	High	Good	Large	High
**Microwave**	>1 mm	Blood, ISF	High	Poor	Portable/wearable	Very Low

ISF, interstitial fluid; OCT, Optical Coherence Tomography.

**Table 4:** Performance comparison of different technologies.

Technology	Experiment	Number of Points	CEG				Sensitivity	*R* ^2^	MAE	LOD
			A	B	C	D				
**NIR** [68]	In vivo	2737	94.2%	5.7%	0%	0.1%	-	-	0.65 mM	-
**MIR** [76]	In vivo	14	100%	0%	0%	0%	-	-	0.67 mM	-
**PA** [100]	In vivo	76	70%	30%	0%	0%	-	-	1.03 mM	-
**Fluorescence** [105]	In vitro	8	-	-	-	-	0.1296 μM^−1^	0.99	-	2 μM
**Raman** [110]	In vivo	34	-	-	-	-	-	0.84	0.37 mM	-
**OCT** [114]	In vivo	81	-	-	-	-	5.78% mM^−1^	0.91	-	-
**THz** [120]	Ex vivo	20	-	-	-	-	-	0.97	0.25 mM	-
**Microwave** [138]	In vivo	89	-	-	-	-	0.0235 dB*mM^−1^	-	-	1.33 mM

MAE, mean absolute error; LOD, limit of detection.
